# Buffalo University

**Published:** 1902-06

**Authors:** 


### Buffalo University.

THE 56th annual commencement of the Department of Medicine and the 15th annual commencement of the department of Pharmacy of the University of Buffalo took place at the Teck Theater Friday morning, May 2, 1902.

At 11 o'clock the members of the faculty of the two departments and the speaker of the morning, the Reverend David J. Burrell, D.D., of the Marble Collegiate Church of New York, took their places on the stage. The Chancellor, the Honorable James O. Putnam, took his appointed place and then the members of the classes in cap and gown filed into their seats, occupying the first four rows in the theater. The exercises were opened first with orchestral selections and then prayer by the Rev. Dr. Burrell. Dr. John Parmenter, secretary of the faculty of medicine, presented the candidates to the chancellor, who conferred the degree of doctor of medicine upon the following-named:

CLASS IN MEDICINE.

Grant Comstock Benjamin, Charles August Bentz, Ernest Grey Bingham, A.B., M.D., James Joseph Brown, Judson Murl Burt, Harry Sefton Campbell, Reger Cutting, Clarence Stanley Hunter Darlington, William Insco Dean, Carl Francis Denman, Charles Frederick Elliott, William Frederick Frasch, Ph. B., Albert Frey, Harrie Van Ness Frink, Robert Ellicott Gray, Charles Haase, Edward E. Haley, Augustus William Hengerer, Eugene Burt Horton, John Braiden Huggins, M. Louise Hurrell, Harry Fisk Hutchinson, James Henry Kellogg, Edward Cox Mann, Thomas Fitzgerald McNamara, Daniel P. Murphy, John Henry Page, Sophy Ellen Page, Richard James Pearson, Arthur Madison Phillips, William Ward Plummer, B. L., Fred Conley Rice, John Raymond Sackrider, George William Seitz, George Nicholas Smith, Otto K. Stewart, Charles Labram Vaux, and Thomas Joseph Walsh.

The honor men were William Frederick Frasch, Ph. B., Eugene Burt Horton, George William Seitz and John Braiden Huggins.

Dr. Matthew D. Mann, dean of the faculty of medicine, administered the Hippocratic oath.

CLASS IN PHARMACY.

Dr. John R. Gray, secretary of the faculty of pharmacy, then presented the candidates from that department and the chancellor conferred the degree of graduate in pharmacy upon the following-named:

Frank May Baldwin, Charles Alfred Bender, Frank Oswald Brickman, David William Briggs, Charles Augustus Brown, Earl Thomas Bryant, Clementine Chapin, Harry B. Ecker, Charles John Engelhardt, Andrew H. Fisk, Wade Edward Gayer, Walter Erastus Gorrie, Michael Moses Harris, Frederick Orrin Henry, Willis Clayton House, Elbridge Gerry Hunton, Lewis Johnson, Lewis Edgar Jones, Thomas Llewellyn Jones, Helena S. Katsmayer, George William Keopka, James Garfield Lloyd, Edward S. Loge, William Harris McCoach, Willis Lee Merkley, Jay Daniel Morton, Vincent Guard Newell, Carl Joseph Nies, Katherine Cooley Quick, Max Charles Salchow, Walter Franklin Sanford, Lucy Cecelia Schorp, Nettie Inez Sheridan, William Lennie Snow, Robert Raikes Street, George E. Swanson, Albert Warren Wagner, Carl George Westling, Harry Martin Wise, Herbert Gladstone Wright and Eugene Rhodes Wolfrom.

Certificates of examination were given to Frank O. Brickman, Charles J. Englehardt, Michael M. Harris, Lewis Johnson, Edward S. Loge, Carl Joseph Nies, Nettie I. Sheridan, Albert

W. Wagner and Carl George Westling, and upon attaining their majority the regular diplomas will be issued to them.

The announcement of honors was made by Dr. Willis G. Gregory, dean of the department of pharmacy.

The William H. Peabody prize of $50 for the highest average standing during senior year, was awarded to Vincent G. Newell.

The four standing next highest in the class in the order given were Andrew H. Fisk, Charles J. Engelhardt, Lewis Johnson and Walter E. Gorrie.

The $25 prize offered by the faculty for the student standing highest in the junior class was awarded to Eda M. Bennett. The next highest were Walter D. Nash, Anna F. Frey, G. Claude Carey and Harlan J. Q. Towe.

### THE DOCTOR'S CREED.

The exercises closed with an address by the Reverend Dr. Burrell, who spoke on The Doctor's Creed. His talk was in the nature of a greeting to the newly-made doctors. He outlined their duties to themselves and to their fellow-men. He said that their work is in the behalf of humanity and it is their duty to answer the cry for help with the tenderest sympathy. He urged them to increase their usefulness to the world by constant study and to work to the end that they shall be not only good physicians, but good men.

### THE ALUMNI ASSOCIATION.

The Alumni Association of Buffalo University Medical College held its 27th annual meeting on University Day, May 2, at which the various exercises as outlined in the JOURNAL for May were held. These included class reunions in the morning, luncheons at midday, a scientific program in the afternoon, and a banquet in the evening.

A full report of this meeting will be published in the next issue of the JOURNAL under the head of Society Proceedings, hence it will be unnecessary to go into detail here. We may add, however, that the meeting was well attended and was one of the most interesting in the history of the association.

### THE CLASS IN DENTISTRY.

The eleventh annual commencement of the department of dentistry was held at the Star Theater, Tuesday afternoon, May 6, 1902, at which the degree of doctor of dental surgery was conferred on 55 candidates, by the Hon. James O. Putnam, chancellor.

The registrar of the faculty, Dr. George B. Snow presented the candidates to the chancellor, who conferred the degree on the following-named:

Dwight Howe Allen, Richard William Barry, Orren Wilson Bates, Arthur DeWitt Becker, George Miller Budlong, Andrew Dexter Cook, Herbert Erastus Cunningham, Frederick Bigelow Dudley, James Roe Dudley, Willard Lee Dutton, John Gibson Elliott, Jesse Lyman Exford, David Fawdrey, DeForest H. Findley, William DeForest Gamble, Frederick Anthony Garvin, Raeside Alexander Germill, William James Graham, Thomas Howard Gray, William Alonzo Griffith, Arthur Stearns Hasbrouck, Frederick Castner Havens, Horace Beals Hawley, Albert Woodsworth Hodges, Eber Little Inman, Ernest Harper Kelsey, Dimmick Enice Lamb, William Henry Leak, George Lewis Leitze, Charles Edward Lewis, James Porter Mallory, Hector Garner Marlatt, John Thomas McIntee, Peter McPherson, Alton De Witt Mesick, Chester Cole Milne, Clifford Frederick Moll, Hubert Alexander Newton, John Donald Ogden, Floyd Robert Roberts, Sarah Martha Schake, Ernest Edmund Schnitzpahn, Hildegarde Elenore Schottky, Raymond Sylvester Scovil, Henry Adolph Semtner, Harold Reynolds Skinner, Byrd Emory St. John, Earle Sheldon Strong, Edward Louis Sugnet, Herbert Walker Taylor, A.B., Emerson Pomeroy Washburn, Aberlt Mitchell Wilbur, Norman Trounce Williams, Henry Clay York and Czar Emmons Zeluff.

Dr. W. C. Barrett, dean, announced the honor men and women, who composed over one-third of the class.

President Rush Rhees, of Rochester University, delivered the annual address to the graduating class and Rev. Dr. W. C. Wilbur, of Hornellsville, spoke on the ethics of professional life.

The annual banquet of the dental alumni association was held at The Genesee Hotel in the evening. A reception in the colonial parlors by the faculty and the graduating class followed the dinner, and the evening ended with dancing.

